# The effect of a rotating magnetic field on the antioxidant system in healthy volunteers - preliminary study

**DOI:** 10.1038/s41598-024-59391-y

**Published:** 2024-04-15

**Authors:** Elżbieta Cecerska-Heryć, Marta Gliźniewicz, Bartłomiej Grygorcewicz, Natalia Serwin, Patrycja Stodolak, Weronika Słodzińska, Radosław Birger, Małgorzata Goszka, Aleksandra Polikowska, Marta Budkowska, Rafał Rakoczy, Barbara Dołęgowska

**Affiliations:** 1 Department of Laboratory Medicine, Pomeranian Medical University of Szczecin, PowstancowWielkopolskich 72, 70-111 Szczecin, Poland; 2 Department of Medical Analytics, Pomeranian Medical University of Szczecin, PowstancowWielkopolskich 72, 70-111 Szczecin, Poland; 3 Department of Chemical and Process Engineering, West Pomeranian University of Technology, Piastów 42, 71-311 Szczecin, Poland; 4 Department of Forensic Genetic, Pomeranian Medical University of Szczecin, Powstancow Wielkopolskich 72, 70-111 Szczecin, Poland

**Keywords:** Rotating magnetic field, SOD, CAT, GPx, TAC, ROMO1, MDA, Oxidative stress, Biochemistry, Biotechnology, Biomarkers

## Abstract

Oxidative stress is characterized by an excessive concentration of reactive oxygen species (ROS) resulting from a disturbance in the balance between ROS production and their removal by antioxidant systems (SOD, CAT, GPx). Prolonged and intense oxidative stress can cause various forms of damage to cells, which markers are total antioxidant capacity (TAC), reactive oxygen species modulator (ROMO1), and malondialdehyde (MDA). It has been demonstrated that magnetic fields can positively affect human health, for example, by reducing oxidative stress. Determination of the effect of a rotating magnetic field (RMF) on the activity/concentration of selected oxidative stress markers. A group of 30 healthy volunteers (15 women and 15 men) (mean age 24.8 ± 5.1) in the study classified into the following groups: internal control group (CG);1 h 25 Hz (samples placed in the field for one hour at 25 Hz); 3 h 25 Hz (samples placed in the field for 3 h at 25 Hz), the 1 h 50 Hz group ( placed in RMF for an hour at 50 Hz), and a group of 3 h 50 Hz (samples placed in the field for 3 h at 50 Hz). Serum samples were collected in K_2_EDTA tubes.. The magnetic induction value obtained for RMF is 37.06 mT and 42.64 mT.Activity/concentration of selected oxidative stress markers was analyzed by ELISA. The influence of an RMF on the activity/concentration of SOD, MDA, TAC, and ROMO1 was demonstrated (p < 0.001; p = 0.0013; p < 0.001; p = 0.003). The RFM can reduce oxidative stress, as evidenced by higher SOD and CAT activities in the CG than in samples placed in the RFM. Prolonged exposure to the RFM at 50 Hz increased the TAC level, indicating an intensification of oxidative stress in these samples. The optimal conditions for staying in the RFM (reducing oxidative stress) are 1 h 50 Hz for SOD and MDA; 3 h 25 Hz for CAT and TAC. In the case of ROMO1, it is stated that 1 h 25 Hz are the optimal conditions for no increased production of ROS.

## Introduction

Reactive oxygen species (ROS) are compounds with an unpaired electron on the valence shell, determining the high reactivity of ions or molecules. This reactivity has a destructive effect on cellular components (carbohydrates, proteins). Therefore, ROS poses a significant threat to the proper functioning of the body^1–3^. The concentration of ROS in the cell should be kept at a constantly low level to maintain homeostasis^4^. Impairment of protective mechanisms by various external influences and pathogenic factors causes a significant increase in the concentration of free radicals in the cell and, consequently, pathological reactions, leading to tissue and cell damage^5^. Cells use the antioxidant protection system to control ROS concentration in the physiological range^6^. The defense mechanisms include enzymes, including catalase (CAT), superoxide dismutase (SOD), and glutathione peroxidase (GPx). The action of antioxidant systems interacts with each other. The substances synergize to reduce the concentration of reactive oxygen species and maintain the balance between antioxidants and oxidants with the constant production of free radicals^7^. This protective system makes it possible to maintain a balance between antioxidants and pro-oxidants. Disturbance of this balance in favour of oxidants is called "oxidative stress^1,2^. Long-term and increased oxidative stress are very harmful to cells^8^. It can lead to inflammation and diseases such as chronic kidney disease, cardiovascular diseases, chronic obstructive pulmonary disease, neurodegenerative diseases, and cancer^9^.

The magnetic field (MF) significantly impacts the human body. Its different frequencies and impact duration can cause harmful and positive effects. The MF regulates biological functions, mainly in the induction/reduction of the inflammatory process, and controls cell differentiation and gene expression^10,11^. It can also increase or decrease the formation of new tissue in vivo, suggesting that the magnetic field is a potential tool to modulate mitotic activity. In addition, previous studies show that the MF effectively counteracts chronic pain^9,10^, reduces oxidative stress^11,12^, and supports cancer therapy (osteosarcoma, breast cancer, gastric cancer, colon cancer, or melanoma)^11^ as a result of reducing proliferation and enhancing apoptosis in various tumour cells. The MF can also affect the properties of the cell: it can change the energy of interatomic and intracellular interactions, it can cause depolarization of cells, and affect the change of pH, coagulation rate, and crystallization rate^12^. Thanks to this, the efficiency of the conducted processes (cell signaling signal transduction or cell differentiation) can be significantly increased^11^. Numerous studies show that MF reduces oxidative stress, supports cancer therapy, and prevents chronic pain^10,11,13^.

This study aimed to analyze the effect of a rotating magnetic field on selected markers of oxidative stress (SOD, CAT, and GPx activity and the concentration of TAC (total antioxidant capacity), ROMO—a modulator of reactive oxygen species), and MDA (malondialdehyde) in healthy volunteers, taking into account various RMF frequency and time spent in the field. Oxidative stress can have serious health consequences as it is the basis for developing certain diseases. Due to the constantly growing number of cancers and neurodegenerative diseases, scientists are trying to find compounds or other factors to reduce oxidative stress. It has been proven that the rotating magnetic field (RMF) effectively reduces it, supporting cancer therapy by reducing proliferation and increasing apoptosis in various cancer cells. Treatment using a rotating magnetic field on living organisms is still in the sphere of ongoing research. It can bring huge benefits and even extend patients' lives, as well as increase the effectiveness of classical therapies^14,15^. The current research is mainly based on determining the harmful effects of the magnetic field on the human body. There are few reports on the possibility of using RMF in medicine. Our study aims to show that RMF can reduce oxidative stress, and its use in medicine (in oncology therapy, treatment of burns, hard-to-heal wounds, or simply supporting traditional therapies) is possible in the future.

## Materials and methods

### Study group

The study group consisted of 30 healthy volunteers (aged 20–45), women (n = 15) and men (n = 15), later in the study classified into the following groups: internal control group (CG); 1 h 25 Hz (samples placed in the field for one hour at 25 Hz); 3 h 25 Hz (samples placed in the field for 3 h at 25 Hz) and the 1 h 50 Hz group (placed in RMF for an hour at 50 Hz) and a group of 3 h 50 Hz (placed in the field for 3 h at 50 Hz) The conditions for exclusion from the study group were chronic diseases declared by the volunteers (e.g., diabetes, kidney disease), infections, operations performed within six months, pregnancy, and contraceptives. In addition, basic biochemical tests were performed in the study group to verify the health status of the study group (glucose, cholesterol, triglycerides, HDL, LDL, albumin, total protein, and creatinine), as well as blood morphology (red blood cell count, platelet count, and leukocyte count). Characteristics of the study group are presented in Tables 1 and 2. Healthy volunteers were informed about the purpose of the study and the possibility of withdrawing from it at any time.

**Table 1:** Characteristic of the study participants (mean ± standard deviation, median—lower and upper quartile).

Parameters	Study group and control group
Sex [M – male, F – female]	M-15/F-15
Age [years]	25 ± 5
	22 (21; 26)
Concentration of creatinine [mg/dl]	0.86 ± 0,19
	0.83 (0,76; 1,02)
Concentration of HDL [mg/dl]	42.47 ± 14.17
	40.23 (33.86; 50.18)
Concentration of albumin [g/dl]	3.93 ± 0.36
	3.88 (3.74; 4.18)
Concentration of total protein [g/dl]	6.35 ± 0.46
	6.30 (6.00; 6.63)
Concentration of cholesterol [mg/dl]	158.75 ± 28.99
	154.42 (136.28; 184.51)
Concentration of glucose [mg/dl]	97.94 ± 7.63
	97.33 (92.00; 101.78)
Concentration of uric acid [mg/dl]	4.51 ± 1.1
	4.47 (3.68; 5.26)
Concentration of TAG [mg/dl]	135.92 ± 25.96
	132.84 (114.43; 153.23)
Concentration of LDL [mg/dl]	97.42 ± 28.60
	94.06 (76.49; 119.90)
Concentration of iron [mg/dl]	68.16 ± 19.49
	70.09 (50.47; 82.24)
White blood cells [x ${10}^{9}/l]$	7.11 ± 2.04
	7 (4; 10.8)
Red blood cells [x ${10}^{12}/l]$	3.86 ± 0.66
	3.75 (3.01; 5.41)
Platelets [μl]	210,533.3 ± 75,773.13
	196,500 (132,000; 441,000)

**Table 2:** Characteristics of the study participants in terms of cigarette smoking, contraceptive use and past surgeries within 6 months.

	N	%
Smoking		
Yes	6	20
No	24	80
Use of contraception		
Yes	3	10
No	14	46.7
Past surgeries within 6 months		
No	27	90
Acute pancreatitis	1	3.3
Mandibular surgery	1	3.3
Marrow donor	1	3.3

*N* number of people.

### Ethical approval and consent

The Bioethical Commission at the Pomeranian Medical University in Szczecin approved the research carried out (no KB-0012/32/2021). All participants, including the healthy volunteers in the control group, were informed about the purpose and scope of the study and gave their consent to donate samples and for the resulting data to be published.

### Samples

**Figure 1:**
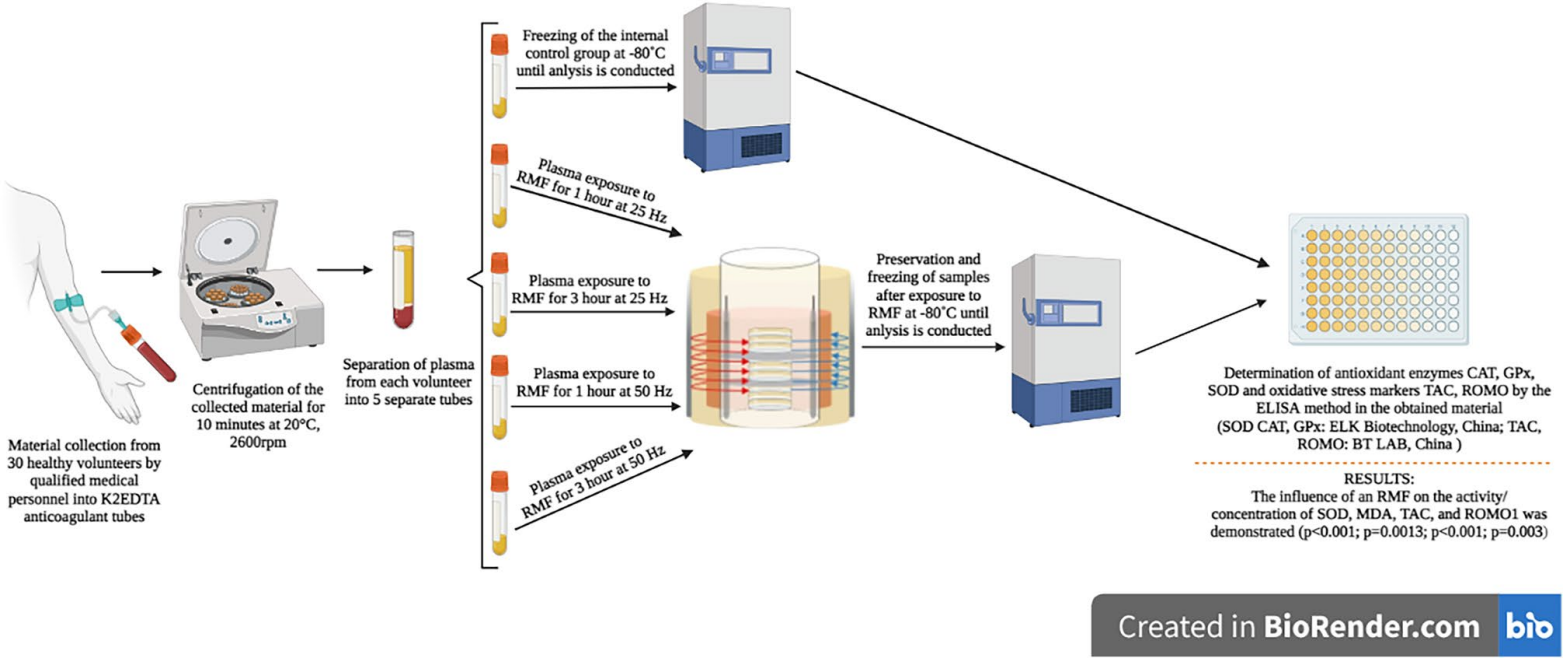
The procedure for collecting biological material and preparing it for further analysis.

The material for the study was plasma, collected once K2EDTA. The plasma was then centrifuged for 10 min at 20 °C, 2600 rpm. Plasma from each volunteer was aliquoted into 5 separate tubes, one of which was an internal control group. The remaining plasma samples, immediately after their preparation, were exposed to a rotating magnetic field at varying frequencies (25 Hz and 50 Hz) for two different time intervals (1 h and 3 h). and inserted into the RMF with different frequencies,using the apparatus located at the Department of Chemical and Process Engineering of the West Pomeranian University of Technology in Szczecin—in two exposure time intervvals.. Plasma from the internal control group was not placed in the RMF. The remaining 4 trials remained at room temperature until the end of the exposure. After this procedure, the plasma was preserved and frozen at – 80 °C. Then, in the collected material, CAT, SOD, GPx, MDA, TAC, and ROMO were marked as measures of oxidative stress. The determination of the tested factors was performed by the ELISA method. The details of this procedure are presented in Fig. 1.

### Rotating magnetic field reactor

**Figure 2:**
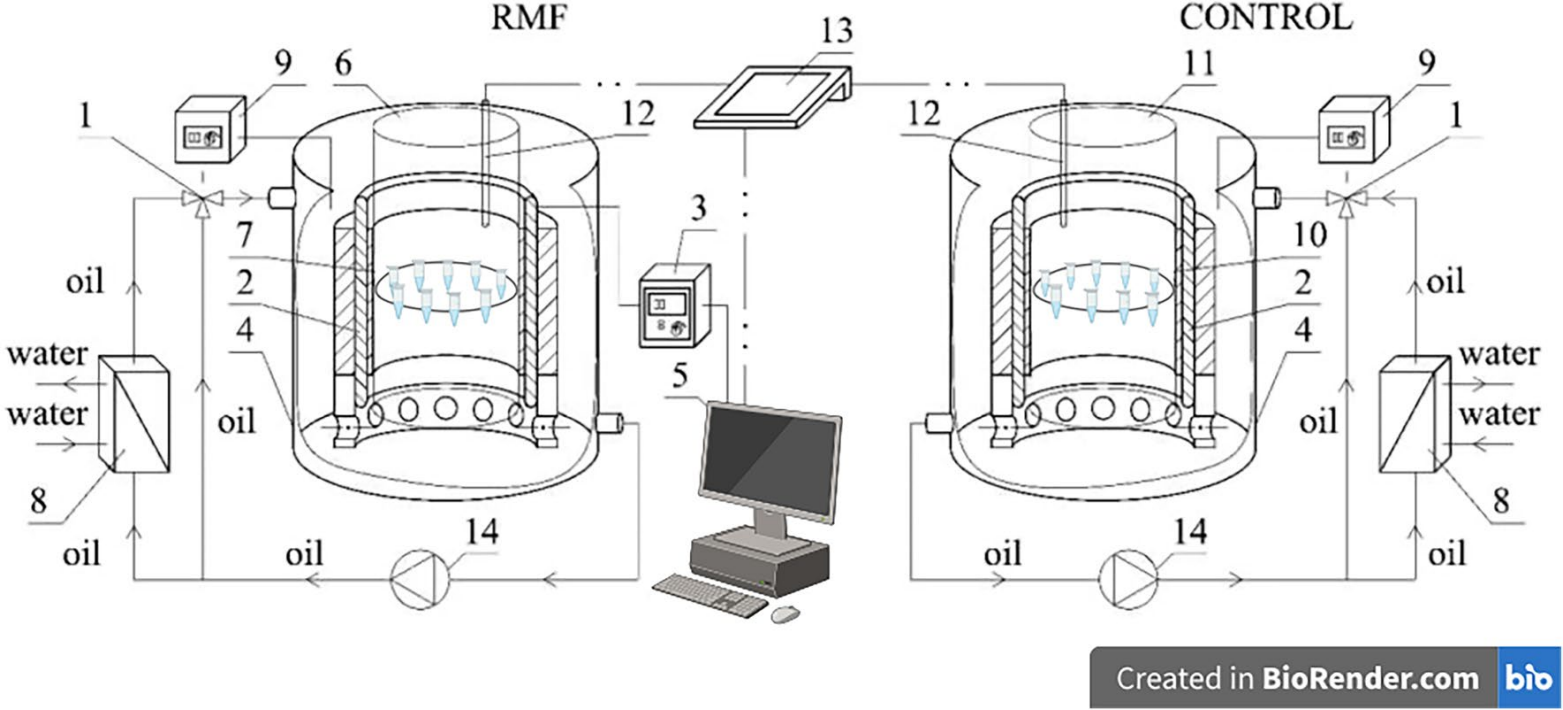
Rotating Magnetic Field application procedure. 1-cooling system; 2-RMF generator; 3-ac tdransistorized inverter; 4-vessel; 5-personal computer; 6—glass container; 7—probe; 8—heat exchanger; 9—thermostat; 10-control probe; 11—control container; 12—temperature sensor; 13—multifunctional computer meters; 14—circulation pump. Figure based on Ref.^34^.

The experimental setup used for this study is presented in Fig. 2. A detailed description of the test apparatus that was used to obtain the results presented in this paper is described in Ref.^16^, and also included in additional materials for the work (Supplementary Materials).

### Activity and concentrations of oxidative stress biomarkers

The activity of CAT, SOD and GPx were each determined by an ELISA (Quantikine^®^ Colorimetric Sandwich ELISAs, ELK Biotechnology, China). The concentrations of TAC and ROMO were determined by an ELISA (Quantikine® Colorimetric Sandwich ELISAs, BT Lab, China; Quantikine^®^ Colorimetric Sandwich ELISAs, ELK Biotechnology, China).

### Statistical analysis

To assess data distributions, the Shapiro-Wilka test was used, which in the case of some variables showed a non-parametric distribution. Exact Fisher and Chi-square tests were used to analyze quantitative data. A Student's t-test and ANOVA were used for univariate systems, and the differences between associated (paired) and unrelated (unpaired) variables were evaluated in the case of variables with a parametric distribution. For variables with non-parametric distributions, a Kruskal–Wallis ANOVA or Friedman ANOVA was used to evaluate differences, as well as the Mann–Whitney U nonparametric test for unpaired data or the Wilcoxon test for paired data. Statistical analysis of the results was carried out using Statistica PL 13 Trial (StatSoft) and R software.

### Ethical approval

All procedures performed in studies involving human participants were in accordance with the ethical standards of the institutional and/or national research committee and with the 1964 Helsinki declaration and its later amendments or comparable ethical standards.

## Results

**Figure 3:**
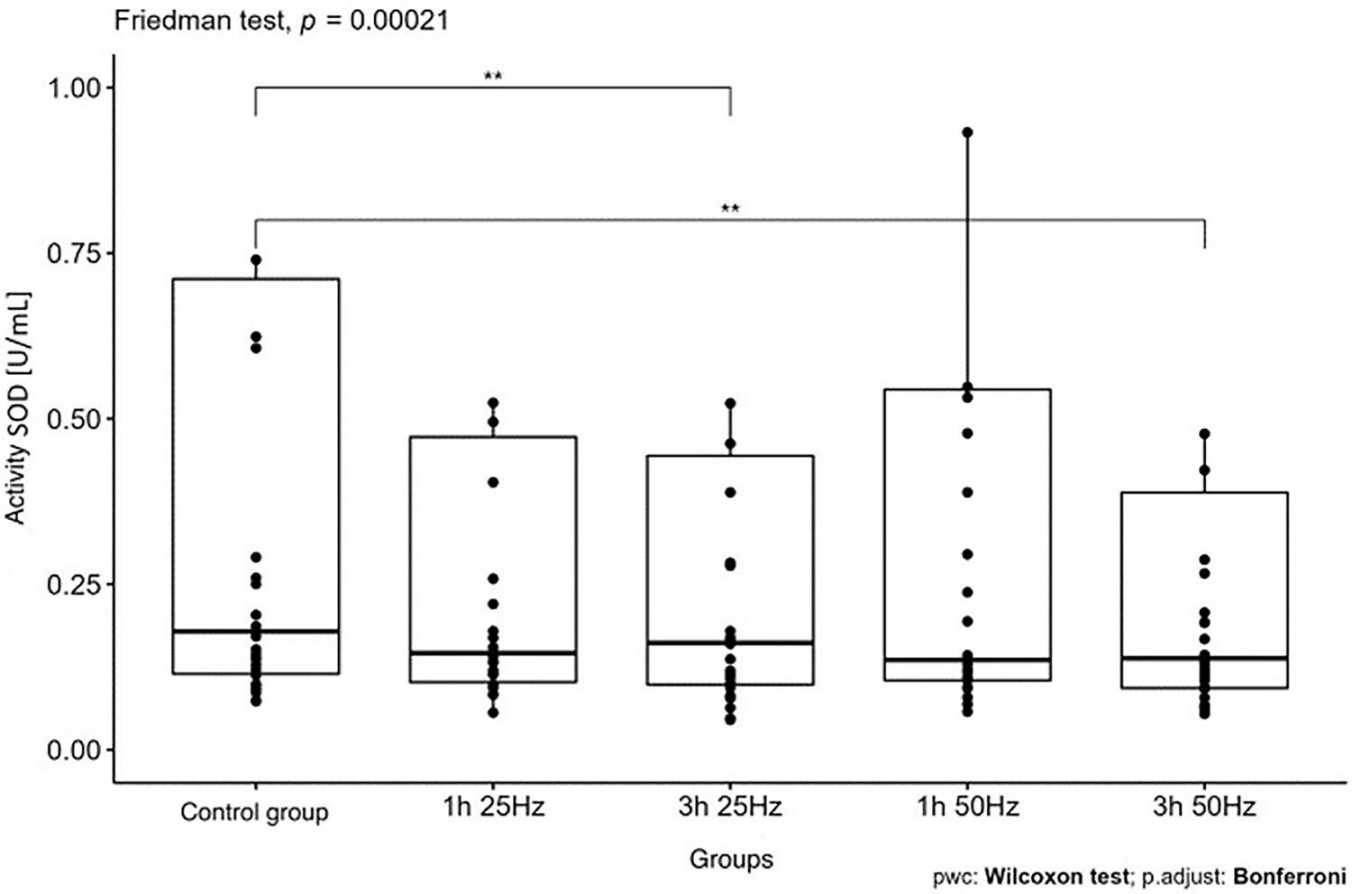
Comparison of SOD activity in different groups [U/mL].

**Figure 4:**
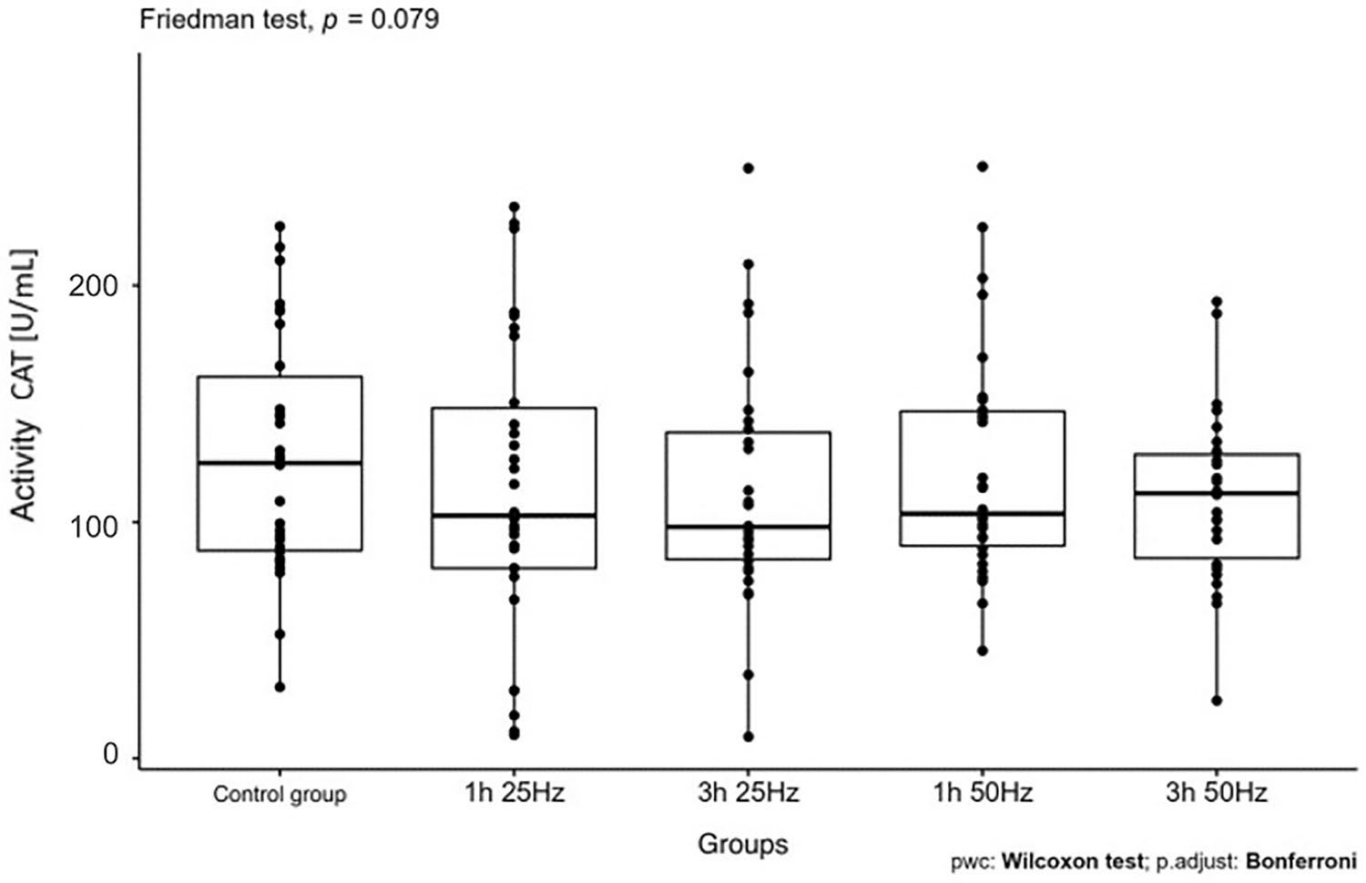
Comparison of CAT activity in different groups [U/mL].

**Figure 5:**
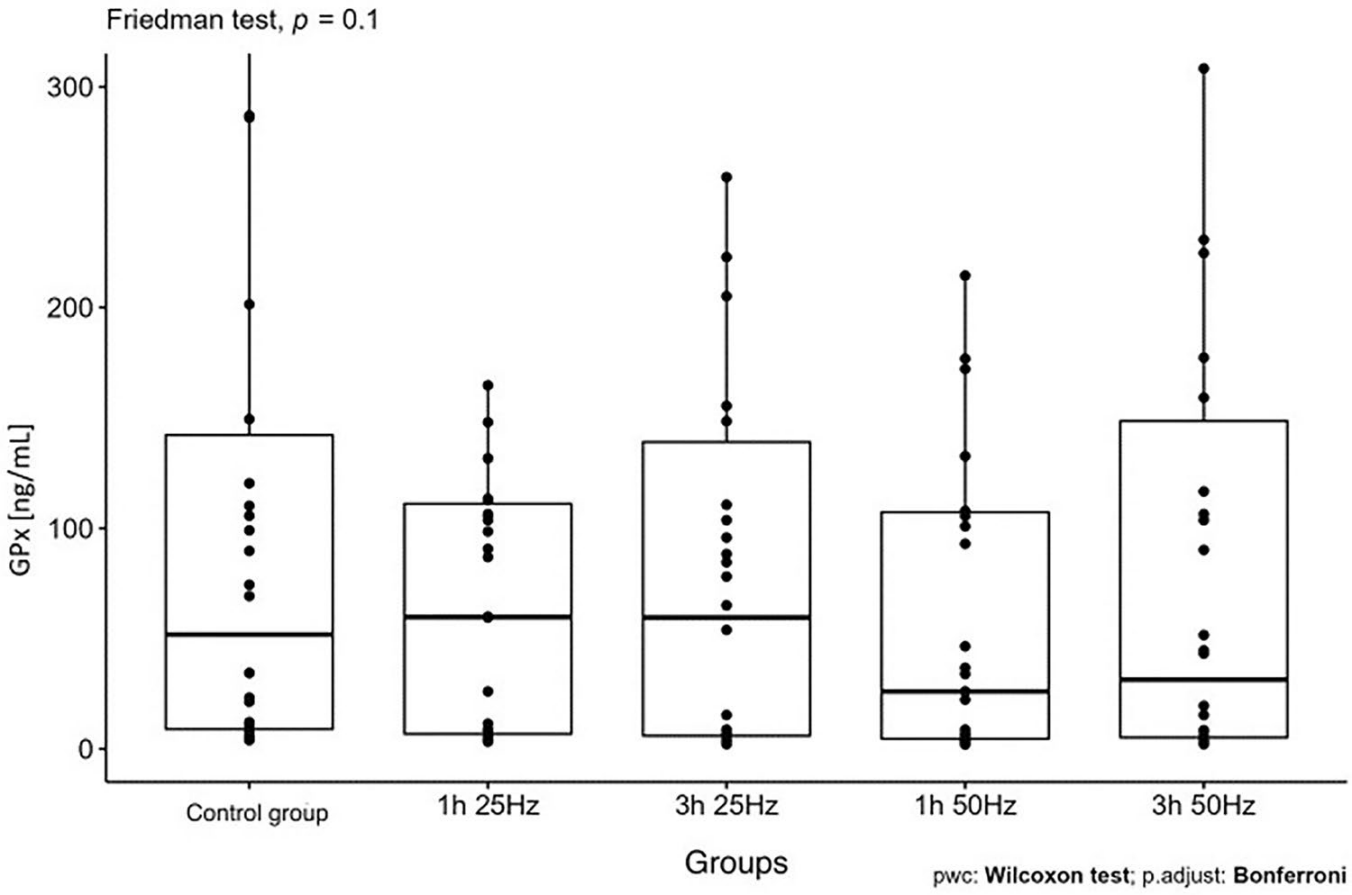
Comparison of GPx concentrations in different groups [ng/mL].

**Figure 6:**
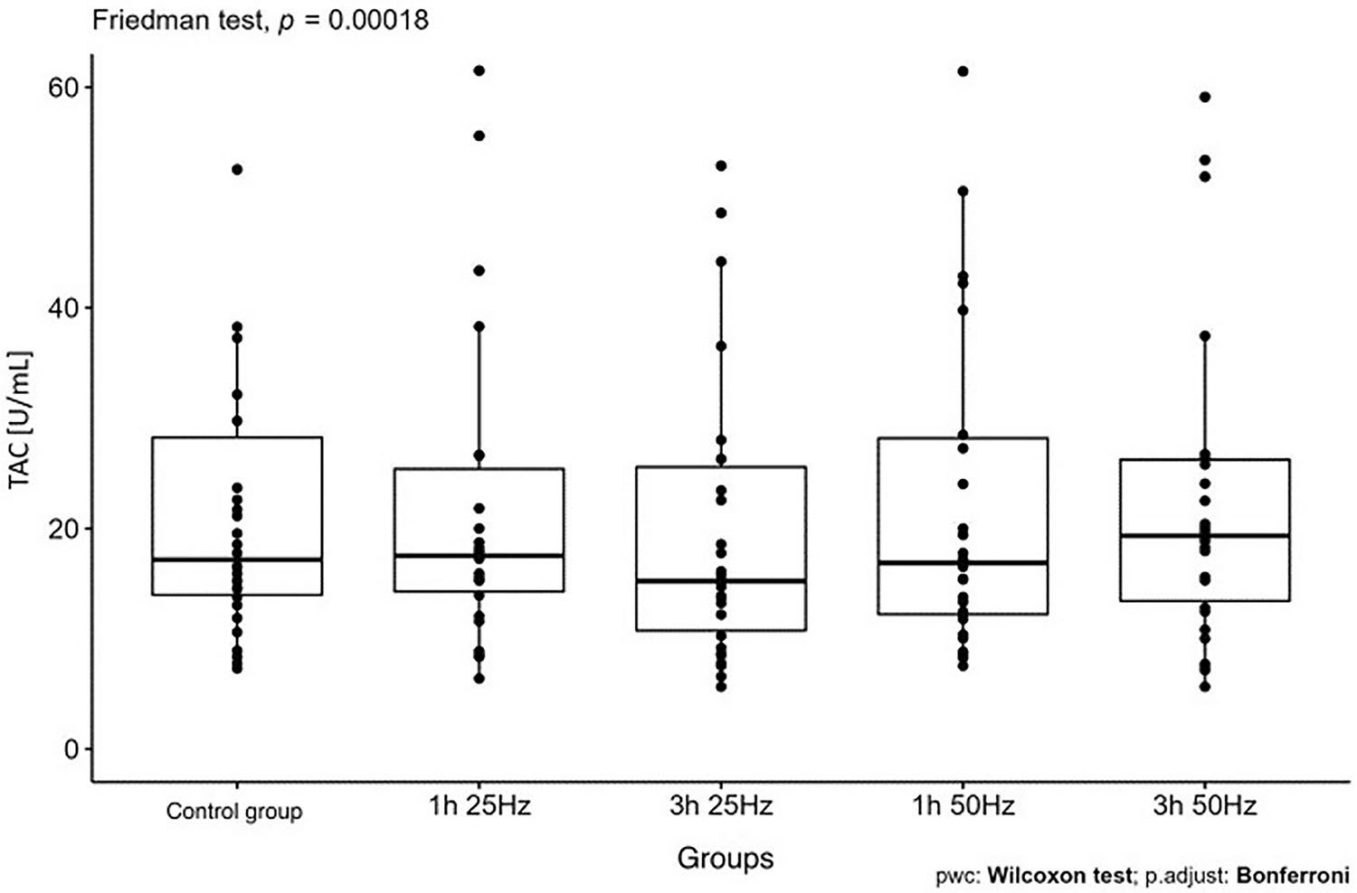
Comparison of TAC activity in different groups [U/mL].

**Figure 7:**
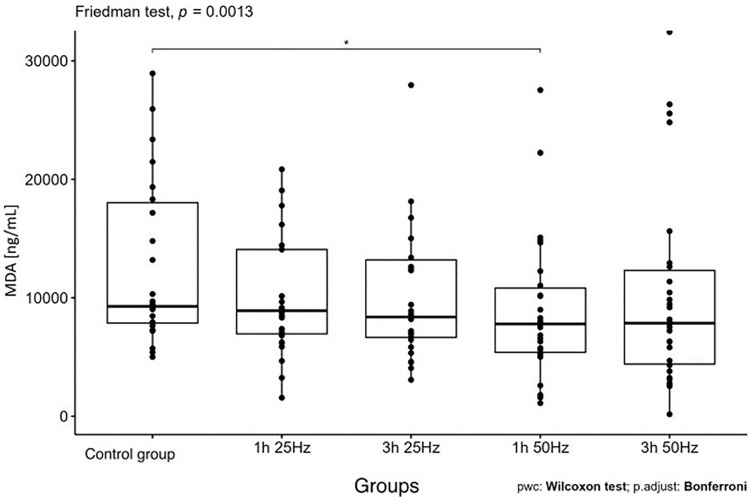
Comparison of MDA concentrations in different groups [ng/mL].

**Figure 8:**
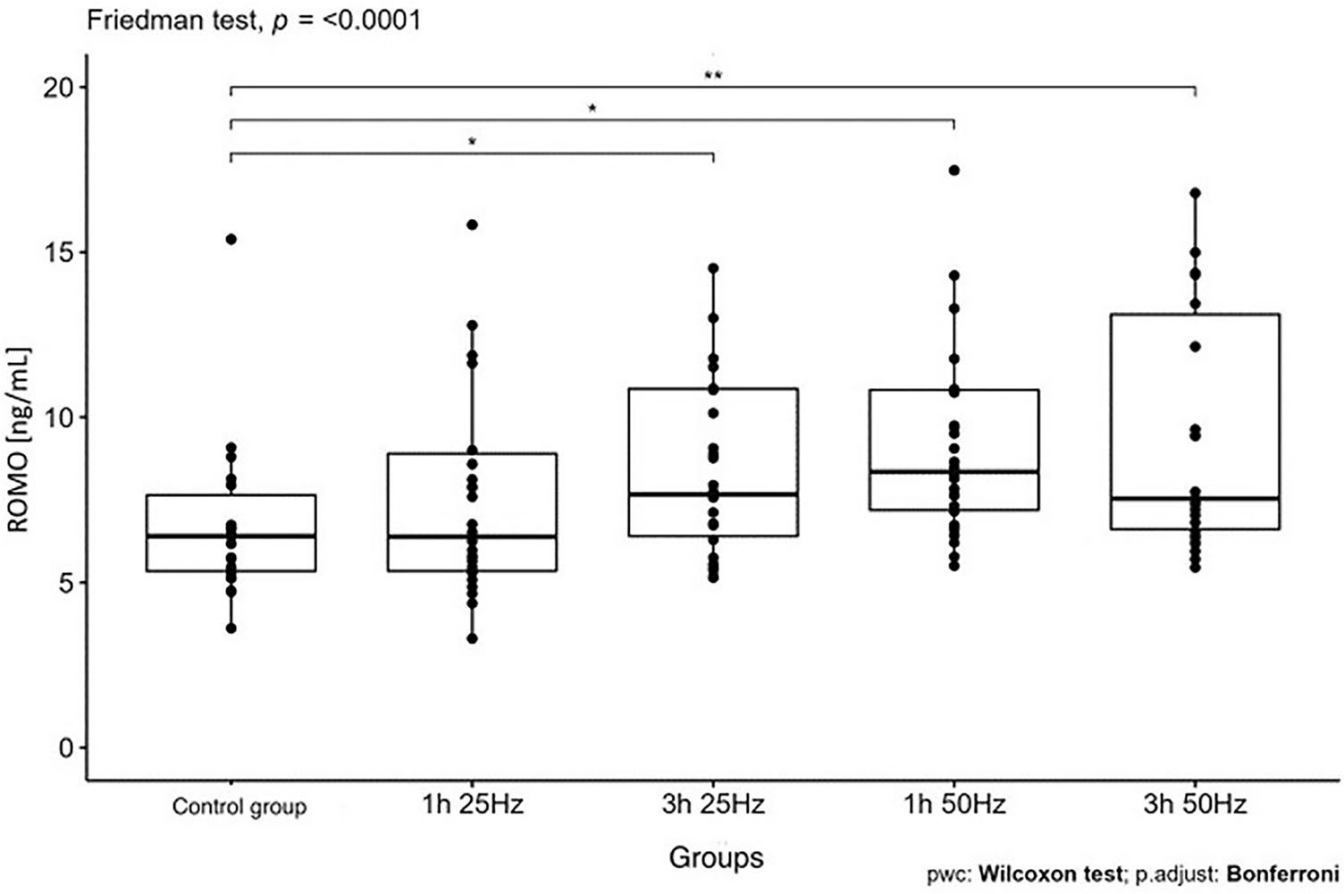
Comparison of ROMO concentrations in different groups [ng/mL].

The influence of the rotating magnetic field (RMF) on the activity/concentration of SOD, MDA TAC, and ROMO1 was demonstrated (p < 0.001; p = 0.0013; p < 0.001; p = 0.003). In the case of CAT, the result was close to statistical significance (p = 0.07). The highest SOD activity was demonstrated in the control group and the samples placed in the field for 3 h 25 Hz. The lowest activity was obtained in samples placed in the field for 1 h at 50 Hz. In the case of MDA, the highest concentration was also shown in the control group and the lowest in samples placed in the field for 1 h at 50 Hz. In turn, the lowest antioxidant capacity was demonstrated in the samples placed in the RMF for 3 h at 25 Hz, and the highest in the 3 h 50 Hz group. In the case of the ROMO1 factor, its highest concentration was found in samples placed in the RMF for 3 h at 25 Hz, and the lowest in the GK and the samples placed in the field for 1 h at 25 Hz (Figs. 3, 4, 5, 6, 7, 8).

Multivariate regression analysis was performed (Table 3). We examined the influence of the group (frequency and time spent in RMF), age, height, and weight (independent variables) on the concentration/activities of oxidative stress markers CAT, MDA, TAC, SOD, GPx, and ROMO (dependent variables). In the case of CAT, the residence time of the RMF and its frequency have been shown to decrease CAT activity by 0.1 U/ml. In the case of TAC, the impact of the whole model on its level was demonstrated in 12%. Age increased TAC activity by 0.4 U/ml. In the case of GPx, the influence of the entire model on its concentration was shown at 21%. Higher age and weight decrease the GPx concentration by 0.24 and 0.58 ng/ml, respectively. In the case of ROMO-1, the influence of the entire model on its level was shown at 13%. The residence time of the RMF and its frequency decreases its concentration by 0.36 ng/ml.

**Table 3:** Analysis of the influence of selected factors on CAT, TAC, SOD activity and MDA, GPx, and ROMO concentration using the multivariate regression method.

	Independent variables	β	R^2^	p	p for model	F
CAT	Age	− 0.10	0.03	NS	NS	2.44
	Growth	− 0.15		NS		
	Weight	− 0.09		NS		
	Group	− 0.10		NS		
MDA	Age	− 0.06	0.01	NS	NS	1.39
	Growth	0.24		NS		
	Weight	− 0.16		NS		
	Group	− 0.04		NS		
TAC	Age	**0.4**	0.12	< 0.001	< 0.001	6.25
	Growth	0.04		NS		
	Weight	0.04		NS		
	Group	− 0.02		NS		
SOD	Age	0.08	− 0.007	NS	NS	0.73
	Growth	− 0.07		NS		
	Weight	0.00		NS		
	Group	− 0.07		NS		
GPx	Age	− 0.24	0.21	0.002	< 0.001	10.76
	Growth	0.27		0.017		
	Weight	− 0.58		< 0.001		
	Group	− 0.01		NS		
ROMO	Group	0.364	0.13	< 0.001	< 0.001	6.81
	Age	− 0.119		NS		
	Growth	0.215		NS		
	Weight	0.067		NS		

Significant values are in bold.

*NS* statistically insignificant, *β* standardized coefficient in the regression equation, *R2* coefficient of determination, *p* significance coefficient value, *CAT* catalase, *SOD* superoxide dismutase, *GPx* glutathione peroxidase, *MDA* malondialdehyde, *ROMO* modulator of reactive oxygen species, *TAC* total antioxidant capacity.

**Figure 9:**
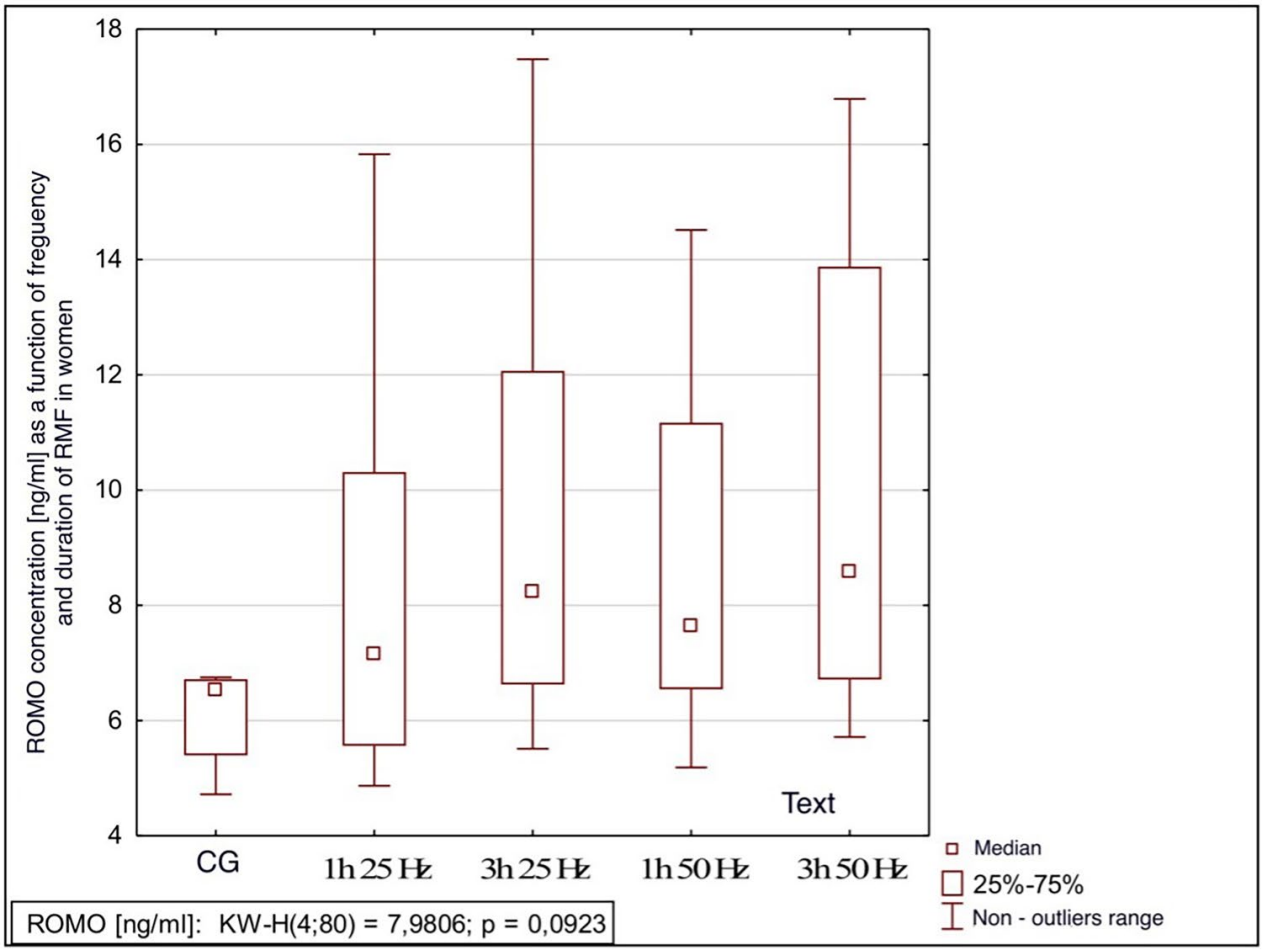
Comparison of ROMO concentrations in different groups in women, p = 0.092 CG-control group; 1 h 25 Hz—plasma samples that were in the field for an hour at 25 Hz; 3 h 25 Hz plasma samples that had been in the field for three hours at 25 Hz; 1 h 50 Hz—plasma samples that were in the field for an hour at 50 Hz; 3 h 50 Hz plasma samples that had been in the field for three hours at 50 Hz.

**Figure 10:**
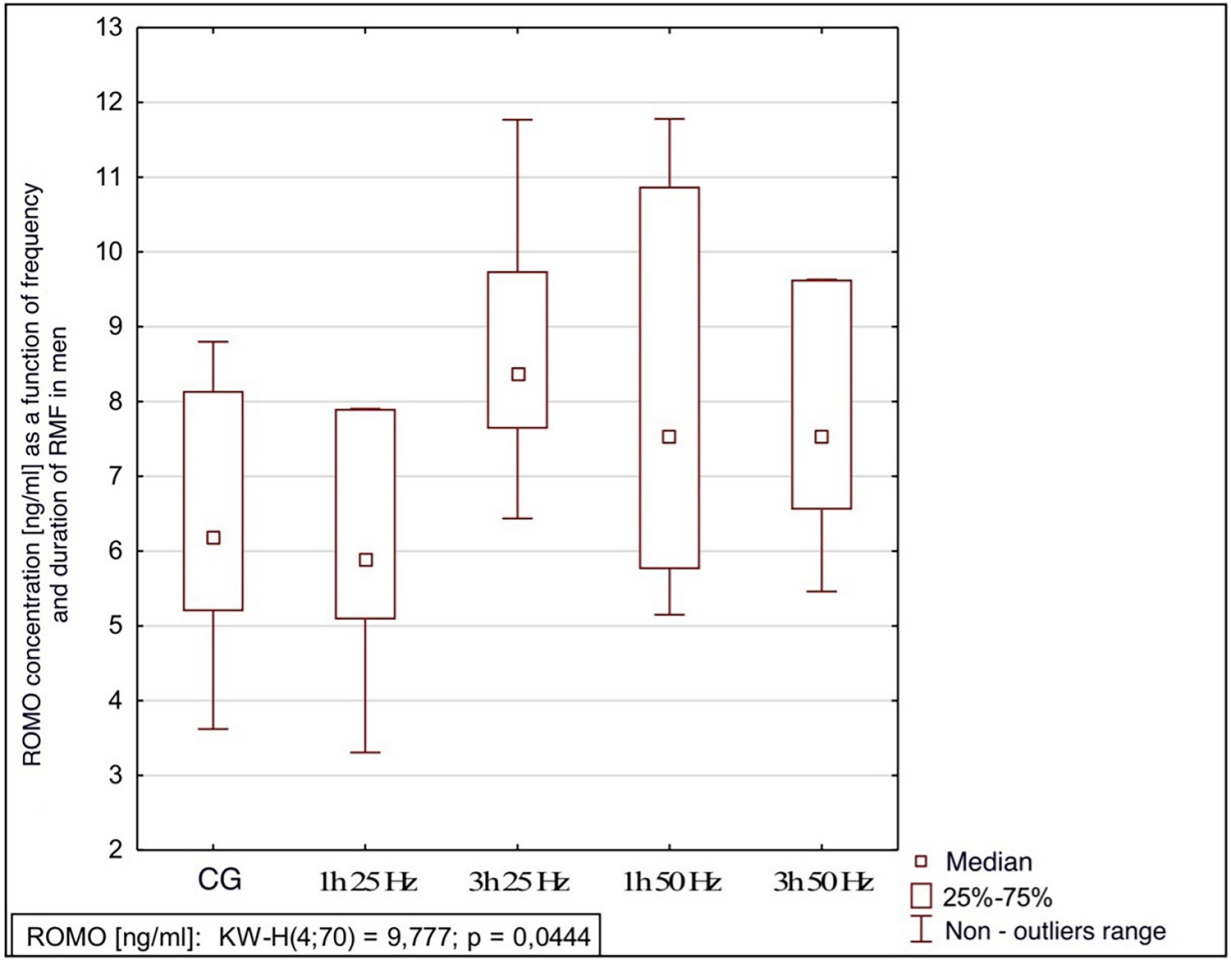
Kruskal–Wallis Rank ANOVA Analysis Comparison of ROMO concentrations in different groups in men, p = 0.044. CG-control group; 1 h 25 Hz—plasma samples that were in the field for an hour at 25 Hz; 3 h 25 Hz plasma samples that had been in the field for three hours at 25 Hz; 1 h 50 Hz—plasma samples that were in the field for an hour at 50 Hz; 3 h 50 Hz plasma samples that had been in the field for three hours at 50 Hz.

It was also shown that the ROMO-1 concentration was influenced by group membership and gender. In the case of men, there was a difference between the concentration of ROMO-1 in particular groups (p = 0.044); in the case of women, there was no such relationship. In men, the highest concentration of ROMO-1 was found in samples that stayed in the field for 3 h at 25 Hz, and the lowest was in samples that remained in the field for 1 h at 25 Hz. At 50 Hz, the residence time was no longer significant. See Figs. 9, 10 for details.

Spearman's rank correlation analysis was performed between the tested markers and enzymes of oxidative stress and sex, age, height, and weight (control group and study groups), the concentration of biochemical parameters: cholesterol, LDL, HDL, triglycerides, albumin, glucose, iron, total protein, creatinine, uric acid (control group), and blood morphology parameters: red blood cell count, platelet count, leukocyte count (control group). Statistically significant correlations are presented in Tables 4, 5, 6, 7, 8, 9. Statistically significant negative, weak correlations (in the study groups and the control group) were found between CAT activity and height and weight (Rs = − 0.166; Rs = − 0.180) and between SOD activity and weight (Rs = − 0.241). A moderate negative correlation was observed between GPx activity and age (Rs = − 0.341). Positive weak correlations were also found between CAT activity and ROMO concentration (Rs = 0.262), between MDA concentration and height and SOD activity (Rs = 0.193; Rs = 0.274), and GPX (Rs = 0.278). And also between TAC concentration and GPx activity (Rs = 0.177), between SOD activity and GPx activity and ROMO concentration (Rs = 0.266; Rs = 0.177; Rs = 0.240). Positive moderate and strong correlations were also observed between CAT activity and SOD and TAC concentration (Rs = 0.475; Rs = 0.365), between TAC concentration and age (Rs = 0.312), SOD activity and ROMO concentration (Rs = 0.382; Rs = 0.683) and between ROMO concentration and age (Rs = 0.311). No statistically significant correlations were observed between the activity of antioxidant defense enzymes, the concentration of oxidative stress markers and blood morphotic elements. The p-values and correlation coefficients are presented in Table 4.

**Table 4:** Spearman's rank correlation coefficients between CAT, SOD, TAC, GPx, ROMO, MDA and studied parameters for the control groups.

CAT	MDA	TAC	SOD	GPx[ng/ml]	ROMO[ng/ml]
Growth	Growth	Age	Weight	Age	Age
Rs = − 0.166	Rs = 0.193	Rs = 0.312	Rs = − 0.241	Rs = − 0.374	Rs = 0.311
p = 0.042	p = 0.018	p < 0.001	p = 0.003	p < 0.001	p < 0.001
Weight	SOD	CAT	CAT	MDA	CAT
Rs = − 0.180	Rs = 0.274	Rs = 0.365	Rs = 0.475	Rs = 0.278	Rs = 0.262
p = 0.027	p < 0.001	p < 0.001	p < 0.001	p < 0.001	p = 0.001
TAC	GPx	SOD	MDA	TAC	TAC
Rs = 0.365	Rs = 0.278	Rs = 0.382	Rs = 0.274	Rs = 0.177	Rs = 0.683
p < 0.001	p < 0.001	p < 0.001	p < 0.001	p = 0.03	p < 0.001
SOD	–	GPx	TAC	SOD	SOD
Rs = 0.475		Rs = 0.177	Rs = 0.382	Rs = 0. 266	Rs = 0.240
p < 0.001		p = 0.031	p < 0.001	p < 0.001	p = 0.003
ROMO	–	ROMO	GPx	–	–
Rs = 0.262		Rs = 0.683	Rs = 0.266		
p < 0.001		p < 0.001	p < 0.001		
–	–	–	ROMO	–	–
			Rs = 0.240		
			p = 0.003		

*Rs* Spearman's rank correlation coefficients, *p* significance coefficient value, *CAT* catalase, *SOD* superoxide dismutase, *GPx* glutathione peroxidase, *MDA* malondialdehyde, *ROMO* modulator of reactive oxygen species, *TAC* total antioxidant capacity.

**Table 5:** Spearman's rank correlation coefficients between CAT, SOD, TAC, GPx, ROMO, MDA and studied parameters for the study groups while staying in a RMF of 25 Hz for 1 h.

CAT	MDA	TAC	SOD	GPx	ROMO
TAC	SOD	CAT	MDA	Age	Age
Rs = 0.403	Rs = 0.421	Rs = 0.403	Rs = 0.421	Rs = − 0.382	Rs = 0.465
p = 0.027	p = 0.02	p = 0.027	p = 0.021	p = 0.037	p = 0.009
–	–	SOD	TAC	–	TAC
		Rs = 0.474	Rs = 0.474		Rs = 0.681
		P = 0.008	p = 0.008		p < 0.001
–	–	ROMO	–	–	–
		Rs = 0.681			
		p < 0.001			

*Rs* Spearman's rank correlation coefficients, *p* significance coefficient value, *CAT* catalase, *SOD* superoxide dismutase, *GPx* glutathione peroxidase, *MDA* malondialdehyde, *ROMO* modulator of reactive oxygen species, *TAC* total antioxidant capacity.

**Table 6:** Spearman's rank correlation coefficients between CAT, SOD, TAC, ROMO, MDA and studied parameters for study groups while staying in a RMF of 50 Hz for 1 h.

CAT	MDA	TAC	SOD	ROMO
Weight	SOD	CAT	CAT	CAT
Rs = − 0.442	Rs = 0.447	Rs = 0.497	Rs = 0.563	Rs = 0.461
p = 0.014	p = 0.013	p = 0.05	p = 0.001	p = 0.01
TAC	–	SOD	MDA	TAC
Rs = 0.497		Rs = 0.395	Rs = 0.447	Rs = 0.907
p = 0.005		p = 0.031	p = 0.013	p = 0.005
SOD	–	ROMO	TAC	–
Rs = 0.563		Rs = 0.907	Rs = 0.395	
p = 0.001		p < 0.001	p = 0.031	
ROMO	–	–	–	–
Rs = 0.461				
p = 0.01				

*Rs* Spearman's rank correlation coefficients, *p* significance coefficient value, *CAT* catalase, *SOD* superoxide dismutase, *MDA* malondialdehyde, *ROMO* modulator of reactive oxygen species, *TAC* total antioxidant capacity.

**Table 7:** Spearman's rank correlation coefficients between CAT, SOD, TAC, GPx, ROMO, MDA and studied parameters for the study groups while staying in a RMF of 25 Hz for 3 h.

CAT	TAC	SOD	GPx	ROMO
SOD	ROMO	CAT	Age	TAC
Rs = 0.440	Rs = 0.703	Rs = 0.440	Rs = − 0.517	Rs = 0.703
p = 0.015	p < 0.001	p = 0.015	p = 0.003	p < 0.001

*Rs* Spearman's rank correlation coefficients, *p* significance coefficient value, *CAT* catalase, *SOD* superoxide dismutase, *GPx* glutathione peroxidase, *MDA* malondialdehyde, *ROMO* modulator of reactive oxygen species, *TAC* total antioxidant capacity.

**Table 8:** Spearman's rank correlation coefficients between CAT, SOD, TAC, GPx, ROMO, MDA and studied parameters for the study groups while staying in a RMF of 50 Hz for 3 h.

CAT	MDA	TAC	SOD	GPx	ROMO
SOD	GPx	SOD	CAT	MDA	Age
Rs = 0.572	Rs = 0.472	Rs = 0.487	Rs = 0.572	Rs = 0.472	Rs = 0.372
p < 0.001	p = 0.008	p = 0.006	p < 0.001	p = 0.008	p = 0.04
–	–	ROMO	TAC	SOD	TAC
		Rs = 0.798	Rs = 0.487	Rs = 0.397	Rs = 0.798
		p < 0.001	p = 0.006	p = 0.029	p < 0.001
–	–	–	GPx	–	–
			Rs = 0.397		
			p = 0.029		

*Rs* Spearman's rank correlation coefficients, *p* significance coefficient value, *CAT* catalase, *SOD* superoxide dismutase, *GPx* glutathione peroxidase, *MDA* malondialdehyde, *ROMO* modulator of reactive oxygen species, *TAC* total antioxidant capacity.

**Table 9:** Spearman’s rank correlation coefficients between CAT, SOD, TAC and GPx, ROMO, MDA and studied parameters in the control group.

CAT	MDA	TAC	SOD	GPx	ROMO
Total protein [g/dL]	Albumin [g/dL]	CAT	CAT	Glucose	LDL
Rs = 0.453	Rs = 0.430	Rs = 0.417	Rs = 0.549	Rs = − 0.388	Rs = 0.396
p = 0.011	p = 0.018	p = 0.021	p = 0.002	p = 0.033	p = 0.03
TAC	–	ROMO	ROMO	–	Total protein[g/dL]
Rs = 0.417		Rs = 0.683	Rs = 0.469		Rs = 0.404
p = 0.021		p < 0.001	p = 0.009		p = 0.027
SOD	–	–	–	–	CAT
Rs = 0.549					Rs = 0.390
p = 0.002					p = 0.032
ROMO	–	–	–	–	TAC
Rs = 0.390					Rs = 0.683
p = 0.032					p < 0.001
–	–	–	–	–	SOD
					Rs = 0.469
					p = 0.009

*Rs* Spearman's rank correlation coefficients, *p* significance coefficient value, *CAT* catalase, *SOD* superoxide dismutase, *GPx* glutathione peroxidase, *MDA* malondialdehyde, *ROMO* modulator of reactive oxygen species, *TAC* total antioxidant capacity, *LDL* low-density lipoprotein.

Considering the study group in which the samples stayed in RMF for 1 h at 25 Hz, statistically significant negative moderate correlations between GPx concentration and age (Rs-0.382) were found. There was also a moderate positive correlation between CAT concentration and TAC activity (Rs = 0.403), between MDA concentration and SOD activity (Rs = 0.421), between TAC concentration and ROMO (Rs = 0.681) and SOD activity (Rs = 0.474), between ROMO and age (Rs = 0.465). The p-values and correlation coefficients are presented in Table 5.

Considering the study group whose samples stayed in the RMF for 1 h at 50 Hz, statistically significant negative moderate correlations between CAT activity and weight were found (Rs = − 0.442). There was a moderate positive correlation between CAT activity and SOD (Rs = 0.563) and TAC and ROMO (Rs = 0.907), between MDA and SOD activity (Rs = 0.421), between TAC and SOD activity (Rs = 0.474). Also, a strong positive correlation was found between TAC and ROMO concentrations (Rs = 0.681). The p-value and correlation coefficients are presented in Table 6.

Considering the study group whose samples stayed in the RMF for 3 h at 25 Hz, statistically significant negative moderate correlations between GPx activity and age were found (Rs = − 0.517). There was a moderate positive correlation between CAT activity and SOD activity (Rs = 0.440). A positive correlation was also shown between TAC and ROMO concentrations (Rs = 0.703). The p-value and correlation coefficients are presented in Table 7.

Considering the study group whose samples stayed in the RMF for 3 h at 50 Hz, statistically significant positive moderate correlations were found between CAT activity and SOD activity (Rs = 0.572), between MDA concentration and GPx activity (Rs = 0.472), between TAC concentration, and SOD activity (Rs = 0.487), between SOD activity and GPx activity (Rs = 0.397), and between ROMO concentration and age (Rs = 0.372). Also, a strong positive correlation between TAC and ROMO concentrations was demonstrated (Rs = 0.798). The p-value and correlation coefficients are presented in Table 8.

Considering the control group, a statistically significant negative moderate correlation between GPx activity and glucose concentration was found (Rs = -0.388). Positive moderate correlations were found between CAT activity and SOD activity (Rs = 0.549) and total protein concentration (Rs = 0.453), TAC (Rs = 0.417), and ROMO (Rs = 0.390). And also between MDA concentration and albumin concentration (Rs = 0.430), between TAC concentration and ROMO concentration (Rs = 0.683), between SOD activity and ROMO concentration (Rs = 0.469), between ROMO concentration and LDL concentration and total protein (Rs = 0.396; Rs = 0.404)). The p-value and correlation coefficients are presented in Table 9.

## Discussion

The study analyzed the effect of the frequency of the rotating magnetic field and the time of its application on the activity of enzymes such as CAT, SOD, and GPx, as well as the effect of RMF on the concentration of TAC (total antioxidant capacity), MDA malondialdehyde) and ROMO1 (reactive oxygen species modulator). Antioxidant enzymes are needed to eliminate the toxic effects of ROS. In many diseases, the activity of these enzymes is reduced, which results in the intensification of harmful changes in the cell. In the conducted study, we determined at what frequencies and at what time the plasma stays in the RMF, the activity of these enzymes increases and decreases. Research using a magnetic field to reduce oxidative stress has been ongoing for many years, but the results still need clarification.

Ciejka et al.^14^ determined the amount of lipid peroxidation (TBARS) in rat muscle tissue. Laboratory animals were divided into 3 groups, depending on the residence time of the samples and the frequency of the alternating magnetic field. In their studies, they showed that a low-frequency magnetic field causes an increase in the concentration of lipid peroxidation products in rat muscle tissue homogenates. The low-frequency magnetic field causes changes in the ROS generation processes. The effect of MF on ROS production has also been demonstrated in other experiments; Lantov et al. and Lupke et al. showed that the generation of reactive oxygen species is also related to the NADPH oxidase activation mechanism^5,17^.

However, Zwirska-Koczala et al. found that MF with a frequency of 200/300 Hz causes increased production of ROS by inhibiting the activity of antioxidant enzymes^18^. ROS are important in physiological processes such as proliferation, growth, differentiation, and apoptosis. However, their overproduction is very unfavourable because it becomes the cause of premature ageing of the body and can lead to many diseases. That is why selecting the parameters used in magnetotherapy is important for the therapy to bring the best results, e.g., in wound healing or cancer treatment.

Superoxide dismutase is the primary antioxidant enzyme. During the dismutation reaction, it removes superoxide anion radicals from the cell. The dismutation reaction transforms two superoxide anion radical molecules in the presence of two protons to H2O2 and O2. Then the toxic hydrogen peroxide is broken down by peroxidase and catalase. With the increase in SOD activity, the concentration of H_2_O_2_ increases, and consequently, the activity of GPx and CAT increases. Disturbed decomposition of hydrogen peroxide and superoxide anion leads to hydroxyl radical, super hydroxyl radical, and singlet oxygen formation. These molecules are the most reactive forms of oxygen. They can lead to cell damage and lipid peroxidation^3,19,20^.

Öztürk et al.^21^ compared 24 cancerous gastric tissues to the same number of non-cancerous tissues also taken from the stomach. The material was obtained during surgical operations. This study showed that static magnetic field (SPM) exerted different effects on oxidative and antioxidant parameters in cancerous and non-cancerous tissues. In the conducted studies, it was found that SPM significantly increases SOD activity in non-cancerous tissue while it decreases SOD activity in cancerous tissue. The various effects caused by SPM may provide valuable information on the adjuvant potential of SPM in cancer therapy, as it can lead to the death of cancer cells by inducing peroxidation reactions in cancer tissues. Different conclusions were drawn by Dudek et al. They showed that the use of phenolic compounds (chlorogenic acid—CGA) as a supportive pharmacological treatment of melanoma is more effective than the simultaneous use of a static magnetic field with chlorogenic acid. Melanoma, control, and CGA-treated cells were placed in extraordinary magnetic test chambers that generated a 0.7 T magnetic field^22^.

RT-qPCR analyzed antioxidant enzyme mRNA levels. SOD, GPx, and CAT activities were measured in cell lysates. The expression and activity of antioxidant enzymes were inhibited in cells treated with CGA (1 mmol/L), in contrast to cells treated with CGA in combination with SMF^22^. However, we must remember that this was a different magnetic field than the one used in our study. Palauszak et al.^23^ studied the effect of MF on the activity of antioxidant enzymes in rats. The study examined the activity of superoxide dismutase, catalase, and hydrogen peroxide in the blood. Laboratory animals were subjected to one-week and two-week exposure to alternating magnetic fields. The authors showed that after a week of application of MF, the concentration of hydrogen peroxide decreased compared to samples in the control group. They also observed decreased CAT and SOD activity. This meant that the magnetic field resulted in a reduction in oxidative stress.

Jiang et al. researched Brassica juncea seeds, considering the magnetic field's influence on life and biochemical processes. So they investigated the impact of the magnetic field on the ability to pick up non-essential elements. To verify this hypothesis, Brassica juncea seeds were treated with fields of 50, 100, 150, 200, and 400 mT, and then the dry matter, cadmium uptake capacity, ferritin content, activity of antioxidant enzymes, and phytoremediation effects of the plant were compared. Compared to the control, low- and medium-intensity fields (50–200 mT) increased plant leaf dry weight by 15.1%, 24.5%, 35.8%, and 49.1%, respectively, while high-intensity fields (400 mT) decreased biomass yield by 18.9%. The Cd content in the aboveground tissues of *B. juncea* increased with the increase in the field strength, accompanied by the increase in oxidative damage. SOD and ascorbate peroxidase (APX) activity increased with exposure to low (50 and 100 mT) and moderate (150 and 200 mT) intensity, followed by a reduction at high intensity (400 mT). Catalase activity (CAT) and ferritin content increased with increasing intensity. This study suggests that the appropriate magnetic field can be considered an environmentally friendly physical trigger to improve the effect of B. Juncewa phytoextraction^24^.

Our study showed a statistically significant difference in SOD activity between the study groups (p < 0.001 depending on the exposure time and RMF frequency). [U/mL] is the lowest, while the highest antioxidant activity was demonstrated in samples placed in the RMF for 3 h at 25 Hz and in the control group. In our study, healthy people were analyzed. Therefore, the reduction in enzyme activity indicates a reduction in the level of oxidative stress compared to their higher concentration in the control group. The change in SOD activity proves the rotating magnetic field's influence on this enzyme's expression. For different times and frequencies, superoxide dismutase activity can be reduced or increased, which can be used in treating various diseases, especially neoplastic diseases.

Catalase is an important enzyme involved in the body's defense mechanisms. It reacts with hydrogen peroxide to form molecular oxygen and water. CAT protects the body against the harmful effects of H2O2^3^. Dalenogare et al.^25^ compared whether the use of Cubiu alone (an Amazonian fruit used as a medicine) has a beneficial effect on skin regeneration processes and whether the use of combined therapy (Cubiu + magnetic field) has a better impact on wound healing. To determine the potential antioxidant and anti-inflammatory properties of cubiu and its interaction with the magnetic field in skin wound healing, skin and blood samples were collected and analyzed on days 3, 7 and 14 of treatment: biomarkers of oxidative stress (thiobarbituric acid reactive substances, non-protein thiols, dismutase superoxide, catalase and glutathione S-transferase), myeloperoxidase enzyme activity and cytokine levels (interleukin 1, interleukin 6, interleukin 10 and tumour necrosis factor-alpha). Cubiu turned out to be safe and non-toxic. Both cubiu and the magnetic field contributed to the reduction of pro-inflammatory and pro-oxidative biomarkers (interleukin 1, interleukin 6, tumour necrosis factor-alpha and thiobarbituric acid reactive substances), as well as the increase of anti-inflammatory and antioxidant biomarkers (interleukin 10, non-protein thiols and dismutase superoxide), with more significant potential when the treatment is used in combination. Catalase activity increased in the Cubiu-treated and the cubiu + magnetic field treatment groups. However, scientists have observed greater regenerative abilities of the skin in combination therapy (Cubiu + magnetic field). Increased activity of the CAT enzyme in a pathological situation is conducive to the greater mobilization of the body to lower ROS.

Benkov et al. studied the therapeutic effects of a simultaneous physiotherapy program that included migrating transcranial magnetic stimulation (TMS) and exposure to low-frequency alternating electrostatic fields (LFEF) in treating patients with metabolic syndrome. Ninety patients were randomly assigned to three study groups. Continuing with usual pharmacotherapy, the first group (30 patients) received the LFEF intervention, the second group (30 patients) received TMS, and the third group (30 patients) received these non-invasive techniques (LFEF + TMS) simultaneously. All treatments included 10 sessions with a daily frequency. Body weight, blood pressure parameters, insulin, cortisol, glucose, total cholesterol, high-density lipoproteins, malondialdehyde, Schiff bases, and the activity of antioxidant enzymes catalase and superoxide dismutase were measured before and after treatment in all patients before and after treatment. Changes in outcomes revealed a different response to LFEF or TMS therapy and a more significant benefit when both medicines were used concomitantly. In particular, the pro-oxidative effect seemed to be significantly inhibited: the level of malondialdehyde decreased by 11.5%, and the level of Schiff bases by 19.3%. At the same time, there was an increase in the activity of antioxidant enzymes (an increase in catalase and superoxide dismutase by approx. 13.2%, respectively). % and 18.6%). This study suggests that the simultaneous intervention of LFEF and TMS is a promising treatment for metabolic syndrome, especially lipid and carbohydrate disorders. However, further studies are needed to confirm these findings^26^.

A fascinating study and similar to the conducted own study, in terms of the frequency of the applied field, although on yeast, was carried out by Lian et al. (ELF-EMF) on prion production and propagation using two budding yeast strains, NT64C and SB34, as a model organism. Under the influence of RF-EMF or ELF-EMF, de novo production and proliferation of yeast prions [URE3] was increased in both strains. The elevation increased over time, and the effects of ELF-EMF were dose-dependent. In addition, ROS levels and SOD and CAT activity were also significantly elevated after short-term exposure (at 0.5, 1.0, and 2.0 h). More prolonged exposure did not cause changes. This work showed for the first time that exposure to EMF can accelerate de novo production and propagation of yeast prions and supports the hypothesis that ROS may play a role in the effect of EMF on protein misfolding and consequently affect cell function^2^.

In our study, the analysis of CAT activity showed a result close to statistical significance (p = 0.079) depending on the exposure time in RMF and the frequency of the field. In my research on healthy people, lowering the level of CAT is beneficial because it indicates a decrease in ROS and, consequently, a reduction in oxidative stress. The most optimal condition for the presence of plasma in RMF for catalase is 3 h at 25 Hz because, under these conditions, catalase had the lowest concentration. In our study, the result was close to statistical significance; increasing the size of the groups would likely cause the statistical significance of the result. However, it should be remembered that in physiological situations GPx has a greater affinity for hydrogen peroxide, and this may be the reason why in the case of CAT, a statistically significant result was not shown^11,20^. Oberley et al.^27^ studied glutathione peroxidase activity in cancer. In his research, he delivered results showing that GPx activity depends on the type of cancer. In the study conducted using Friedman's test, the analysis of GPx activity did not show statistical significance (p = 0.1). The lowest concentration of GPx was found when the plasma was in the RMF for 1 h and the 50 Hz, while the highest activity of glutathione peroxidase was found during the stay in the RMF for 3 h at the 25 Hz and in the control group. The study did not show statistical significance; it may depend on the fact that the rotating magnetic field has a more significant effect on enzymes belonging to the group of metalloenzymes. On the other hand, metalloenzymes include, above all, superoxide dismutase.

Inflammatory markers are biological indicators used to confirm the presence of pathologies and disease states. The most important markers include MDA (malondialdehyde) and TAC (total antioxidant capacity), which is a marker of the ability to "scavenge" reactive radicals. The TAC assay is used to assess the antioxidant potential of the body's cells and can be used together with some markers of inflammation to determine disease activity^28^. Feng et al.^29^ analyzed the effect of a static magnetic field on the reduction of oxidative stress while examining wound healing and diabetic complications. The study compared the ways of wound healing in healthy mice and mice with diabetes. Researchers in the study showed that SPM causes an increase in the level of SOD and a decrease in the level of MDA in the wound tissues of diabetic mice. Summing up the research, Feng et al. showed that SPM could reduce ROS and oxidative stress, reducing the MDA level and consequently promoting wound healing in diabetic mice. In turn, Akdag et al. showed that long-term exposure to 100 μT and 500 μT ELF-MF did not affect oxidative or antioxidative processes (CAT, TAC, TOS, and OSI), lipid peroxidation (MDA) or reproductive elements such as spermatozoa and morphology in rat testis tissue^30^. Sieroń et al.^8^ studied the impact of electromagnetic fields (low frequency—LF-EMF; frequency: 50 Hz; intensity: 10 kV/m; magnetic induction: 4.3 pT; radio frequency (RF-EMF) emitted by mobile phones (frequency: 900 MHz) on the pro-oxidant and antioxidant balance in rat gastrointestinal homogenates After 28 consecutive days, the following levels of SOD and its two isoenzymes (Mn-SOD, Cu, Zn-SOD), CAT, GPx, GR, GST, total antioxidant capacity (TAC), total oxidative potential (TOS) and malondialdehyde (MDA). This study showed that low-frequency electromagnetic fields caused the most significant disturbance of oxidative stress in the rat digestive tract^12^. In our study, the effect of frequency and time in the RMF to MDA concentration (p < 0.001) The highest concentration was found in the control group, and the lowest concentration in samples placed in the field for 1 h at 50 Hz. Under the same conditions, the highest SOD activity was achieved, confirming that 1 h and 50 Hz reduce oxidative stress and ensure the results obtained by Lian et al. They indicated that the increase in SOD activity occurs at low field frequencies and within a short time of its application. This proves the influence of RMF on MDA activity^2^. Thanks to the use of different frequencies and different residence times of the plasma in the MF, the activity of MDA can be influenced. This brings beneficial effects in treating certain diseases, and it has been shown that reduced MDA activity accelerates the wound-healing process.

Our study demonstrated the influence of the frequency and time of residence of plasma samples in RMF on TAC activity (p < 0.001). The highest TAC activity was shown in examples placed in RMF for 3 h at 50 Hz. In contrast, the lowest activity was found in samples placed in the field for 3 h at 25 Hz. This shows that frequency, not time in the field, significantly impacts the TAC. These results are also consistent with those achieved with SOD or MDA. They confirm that the lowest oxidative stress accompanies samples placed in the field for 3 h at 25 Hz.

Reactive Oxygen Species Modulator 1 (ROMO1) is a determining protein found in the inner membrane of the mitochondria. ROMO1 stimulates the production of ROS in the mitochondria, especially in cells with a high metabolism. This protein is associated with several key proteins and signalling pathways, especially the TNF-α and NF-κB pathways. These pathways play a crucial role in oxidative stress and inflammation. For this reason, it is assumed that ROMO1 may directly or indirectly induce oxidative stress^20^. There are no reports in the literature on the influence of the electromagnetic field on ROMO1. However, other studies show that the ROMO factor can be a very interesting marker of oxidative stress because it informs about the formation of ROS. Kim et al.^31^ showed that overexpression of the ROMO1 factor is significantly correlated with early recurrence and unfavourable prognosis in non-small cell lung cancer patients. ROMO1 is associated considerably with VEGF-C, suggesting that the ROMO1 factor may cause lymphoid metastasis through increased ROS generation. Therefore, the ROMO1 factor can be used as a prognostic marker of inflammation. Research is underway to make ROMO1 a therapeutic target to regulate inflammation. However, Amini et al.^32,33^ dealt with the relationship between COVID-19 and the concentration of the ROMO-1 factor. According to research, COVID-19 may promote oxidative stress through some important pathways, for example, the TNF-α and NF-κB pathways. In addition, ROMO1 is closely linked to these pathways, and its dysfunction can affect them, promote oxidative stress, and ultimately cause tissue damage, especially in the lungs. Another factor to consider is that the TNF-α and NF-κB pathways are related to ROMO1, COVID-19, and oxidative stress. In conclusion, it is hypothesized that COVID-19 may increase oxidative stress by affecting ROMO1. Based on this, the researchers concluded that understanding the exact molecular mechanisms of ROMO1 in the pathogenesis of COVID-19 could pave the way to finding better therapeutic strategies.

Our study showed the effect of the frequency and time spent in the RMF on the concentration of ROMO1 (p < 0.001). Its highest concentration was found in samples placed in the RMF for 1 h at 50 Hz. The lowest concentration was found in the control group, and the samples were placed in the field for 1 h at 25 Hz. This shows that, as in the case of TAC, it is the frequency, not the time spent in the field, that significantly impacts the concentration of ROMO1. A lower frequency with the same sample residence time in the field causes a decrease in the concentration of the ROMO1 factor. It is a very good prognostic marker because lowering its concentration leads to a reduction in the concentration of ROS.

As a consequence, the parameters of oxidative stress will decrease. It can be used in the treatment of certain diseases, as well as increase the effectiveness of definitive therapies. Based on the analyzed results, the frequency of 25 Hz is the most optimal for healthy people.

Despite the very promising research results obtained in our experiment, it should be taken into account that the results obtained are the results of preliminary research and require confirmation on a larger study group.

## Conclusion

The rotating magnetic field may reduce oxidative stress, as evidenced by higher activities/ concentrations of SOD, CAT, or MDA in the internal control group than in the samples placed in the RMF. Too long a stay in the RMF at the frequency of 50 Hz increased the level of TAC, which proves the increase of oxidative stress in these samples. The optimal conditions for the plasma to stay in the RMF (reducing oxidative stress) are 1 h and 50 Hz for SOD and MDA; 3 h 25 Hz for CAT and TAC. On the other hand, in the case of ROMO1 (whose overexpression proves the intensification of oxidative stress), they indicate that 1 h 25 Hz are optimal conditions in which there is no increased production of reactive oxygen species. Frequency, not time in the field, significantly impacts TAC activity. These results are also consistent with those achieved with SOD or MDA. They confirm that the lowest oxidative stress accompanies samples placed in the field for 3 h at 25 Hz. However, it should be emphasized that the obtained results are the results of preliminary studies and require confirmation from a larger study group (Supplementary Information).

### Supplementary Information

Supplementary Information.

## Data Availability

All data generated or analysed during this study are included in this published article.
