# Supplementary material for: The effect of a rotating magnetic field on the antioxidant system in healthy volunteers - preliminary study
Source: Sci Rep. 2024 Apr 15;14:8677. doi: 10.1038/s41598-024-59391-y (PMC11018782; doi:10.1038/s41598-024-59391-y)
Supplement: Supplementary file 1 — Supplementary Information. [file 41598_2024_59391_MOESM1_ESM.docx]

The experimental system used in this entry is shown in Figure 1. This apparatus comprises the system (1) and the RMF generator (2). The presented research generated RMF by a three-phase stator equipped with a set of coils (windings) taken from a squirrel-cage motor. The stator consists of three sets of coils powered by each phase and characteristic elements around one axis every 120°. Current flows through a coil, producing electromagnetic fields that interfere with others, followed by superposition provided by RMF fields. The RMF created is disconnected from an external vector that gives direction in time (rotating around an axis), but the output field is constant. However, the fields may change with distance: they decrease from the coils to the central generator in the horizontal direction. There may be a change in the distance from the vertical height. The RMF generator (2) has a transistor AC inverter (3). The inverter used allows you to set the frequency in the AC network. Output frequency (output frequency between the inverter output terminals) and output output (voltage between the inverter output terminals) can be adjustable parameters. The RMF value generated in the system by startup, after enabling the option, is in proportion to the field frequency value. The inverter is connected to a computer (5) equipped with software controlling all current parameters and the RMF frequency. Following the tests, the output result is a current of 25 Hz (maximum induction of the operating magnetic field Bmax = 45 mT). The temperature of the entire setup (in the medium) is controlled by the setup setup (1), which is equipped with a Pt-100-type sonic temperature. As the RMF generator produces heat through the power coils, it controls the rise in temperature of the thermostat (9).

Typical oil from the generator tank was pumped from the bottom to the top. When the temperature spread, the oil passed through the plate heat exchanger fed by tap water, lowering its temperature. Possibility to adjust the configuration and establish which results in stabilized conditions at 37 ± 0.2°C. Temperature fluctuations inside glass containers (6) and controllers (11) were measured using temperature controllers (12) devices with multifunctional computer devices (13). Magnetic induction values at various locations inside the cylindrical chamber were measured using a transverse Hall probe (STD18-0404) connected to a G-meter (FW Bell 5,180 Magnetometer G; Magnetic Science Inc., United States) with a measurement measurement of ± 2.5% of the reading. Measurements of magnetic induction in the active stator ("stator tooth").

Final results methodology in supplementary materials (Supplementary Section S1.2, Supplementary Results S2–S8). The magnetic induction value obtained for RMF is 37.06 mT and 42.64 mT for the output current frequencies f = 25 Hz and f = 50 Hz, respectively. The obtained values of magnetic induction at different points inside the RMF generator are presented in the form of patterns of magnetic induction. **Figure S1** shows the contour patterns of the spatial distributions of the magnetic field in the selected cross-section of the RMF generator. It should be noticed that the presented patterns of the magnetic induction were obtained for the frequencies of the electrical current equal to 25 and 50 Hz. The experimental procedure was carried out by using these frequencies of the electrical current. Based on the investigations of the magnetic in-duction, the maximal values of this parameter, B_max_, might be obtained. It should be noticed that the maximum values of magnetic induction are in the area near the RMF generator. The values of the magnetic induction are decreased towards the centre of the RMF generator. As follows from the analysis of the experimental data, the values of magnetic induction are spatially distributed in the volume of the RMF generator. Therefore, the applied RMF might be characterized employing the averaged values of magnetic induction.


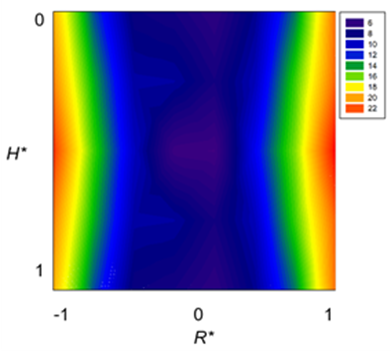

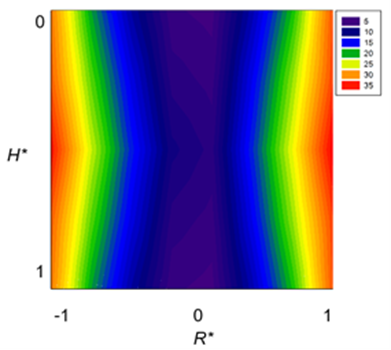


1. **(b)**

**(c)**

**Figure S1.**The contour pattern of the spatial distribution of the magnetic induction in the selected cross-section of the magnetically assisted photoreactor (MAP) for the frequency of the electrical current equal to:**(a)** 25 Hz and **(b)** 50 Hz.
